# Gabaergic Interneurons in Early Brain Development: Conducting and Orchestrated by Cortical Network Activity

**DOI:** 10.3389/fnmol.2021.807969

**Published:** 2022-01-03

**Authors:** Davide Warm, Jonas Schroer, Anne Sinning

**Affiliations:** Institute of Physiology, University Medical Center of the Johannes Gutenberg University, Mainz, Germany

**Keywords:** cortex, development, activity patterns, interneuron, GABA shift, apoptosis, migration, synaptogenesis

## Abstract

Throughout early phases of brain development, the two main neural signaling mechanisms—excitation and inhibition—are dynamically sculpted in the neocortex to establish primary functions. Despite its relatively late formation and persistent developmental changes, the GABAergic system promotes the ordered shaping of neuronal circuits at the structural and functional levels. Within this frame, interneurons participate first in spontaneous and later in sensory-evoked activity patterns that precede cortical functions of the mature brain. Upon their subcortical generation, interneurons in the embryonic brain must first orderly migrate to and settle in respective target layers before they can actively engage in cortical network activity. During this process, changes at the molecular and synaptic level of interneurons allow not only their coordinated formation but also the pruning of connections as well as excitatory and inhibitory synapses. At the postsynaptic site, the shift of GABAergic signaling from an excitatory towards an inhibitory response is required to enable synchronization within cortical networks. Concomitantly, the progressive specification of different interneuron subtypes endows the neocortex with distinct local cortical circuits and region-specific modulation of neuronal firing. Finally, the apoptotic process further refines neuronal populations by constantly maintaining a controlled ratio of inhibitory and excitatory neurons. Interestingly, many of these fundamental and complex processes are influenced—if not directly controlled—by electrical activity. Interneurons on the subcellular, cellular, and network level are affected by high frequency patterns, such as spindle burst and gamma oscillations in rodents and delta brushes in humans. Conversely, the maturation of interneuron structure and function on each of these scales feeds back and contributes to the generation of cortical activity patterns that are essential for the proper peri- and postnatal development. Overall, a more precise description of the conducting role of interneurons in terms of how they contribute to specific activity patterns—as well as how specific activity patterns impinge on their maturation as orchestra members—will lead to a better understanding of the physiological and pathophysiological development and function of the nervous system.

## Introduction

During early development, mammalian brains can be functionally characterized by the progressive emergence of distinct cortical activity patterns which are essential for the establishment of basic functions of the cerebral cortex (Blankenship and Feller, 2010; Kilb et al., 2011; Kirkby et al., 2013). Underlying this dynamic change in neuronal activity, among other developmental processes, is the structural and functional maturation of the two main signaling principles of neurons: excitation and inhibition (Egorov and Draguhn, 2013; Luhmann et al., 2016; Teppola et al., 2019). While glutamatergic signaling is established already within early, embryonic stages in rodents and humans (Monyer et al., 1994; Bagasrawala et al., 2017), the maturation of the GABAergic system extends into the postnatal period of most mammals. Starting with the formation of the first GABAergic synapse (Khazipov et al., 2001), the maturation of the inhibitory system coincides with the emergence of correlated oscillatory activity patterns, such as spindle burst and gamma oscillations in newborn rodents or delta brushes in prenatal humans (Luhmann and Khazipov, 2018). Here, the thalamus contributes significantly to the generation of these early cortical oscillations (Minlebaev et al., 2011; Yang et al., 2013; Murata and Colonnese, 2016), which conversely also modulate thalamic activity within a cortico-thalamic feedback loop (Yang et al., 2013; Martini et al., 2021). Furthermore, the maturation of the adult inhibitory GABAergic system is still not complete when cortical activity evolves to its final more de-correlated activity state that underlies its later complex functions (Golshani et al., 2009; Rochefort et al., 2009).

Although the contribution of GABA signaling to cortical activities during the perinatal phases is not fully understood, it is often speculated that GABAergic interneurons critically modulate the output of neuronal circuits in the form of spontaneous and sensory-driven activity (Bonifazi et al., 2009; Butt et al., 2017). In general, the importance of distinct cortical activity patterns during cortical development is emphasized by their necessity for and coherent emergence with higher cognitive function (Tort et al., 2009; Bosman et al., 2012). Consistently, in sensory cortical areas during the postnatal period of rodents, stereotypical spontaneous and evoked activity patterns concurrently develop with respective sensory modalities (Rochefort et al., 2009; Yang et al., 2009; Colonnese et al., 2010; Ackman and Crair, 2014; Martini et al., 2021). However, the source of spontaneous activity is still a matter of ongoing research, as well as the root cause and type of evoked activity which varies depending on the region and time point of perinatal development.

Yet, undoubtedly, neuronal activity itself is a key regulator of many—if not all—developmental processes in the cortex. Thus, it comes to no surprise that neuronal activity also strongly impacts the maturation of the GABAergic system, from the cellular to the network level, and fine-tunes excitation/inhibition balance (Turrigiano and Nelson, 2004; Takesian and Hensch, 2013). Besides cortical activity, thalamic inputs also play a role in interneuron maturation on the level of the cortex (Pouchelon et al., 2014; Marques-Smith et al., 2016), while interneurons in turn function as a gate for the thalamus by effecting cortical network activity (Yang et al., 2013; Martini et al., 2021). Therefore, the GABAergic system must permit sufficient excitation to engage in cortical activity and still provide the needed inhibition to prevent over-excitation. In this respect, it is worth mentioning that a certain level of freedom in the excitation/inhibition balance is needed, especially for the establishment of the sensory cortical system during early development (Masquelier et al., 2009; Deidda et al., 2015; Wosniack et al., 2021). Both the GABAergic system and neuronal activity are fulfilling important functions during these critical periods, as discussed in more detail elsewhere (Sale et al., 2010; Reha et al., 2020).

In this article, the focus will be on the interdependency of the maturation of the GABAergic system and cortical network activity throughout the perinatal and postnatal development of the rodent cortex. For this purpose, we will review how neuronal activity impacts the maturation of the GABAergic system on the subcellular (mostly synaptic) level, on the cellular and on the network level and discuss how the maturation on each of these scales feeds back on cortical activity, thus impacting the function of the mature cortex. Finally, we will describe the physiological implications of this interdependency and highlight open questions in this field of research.

## The Interplay of Activity and Perinatal Changes of GABAergic System at The Subcellular Level

**Figure 1:**
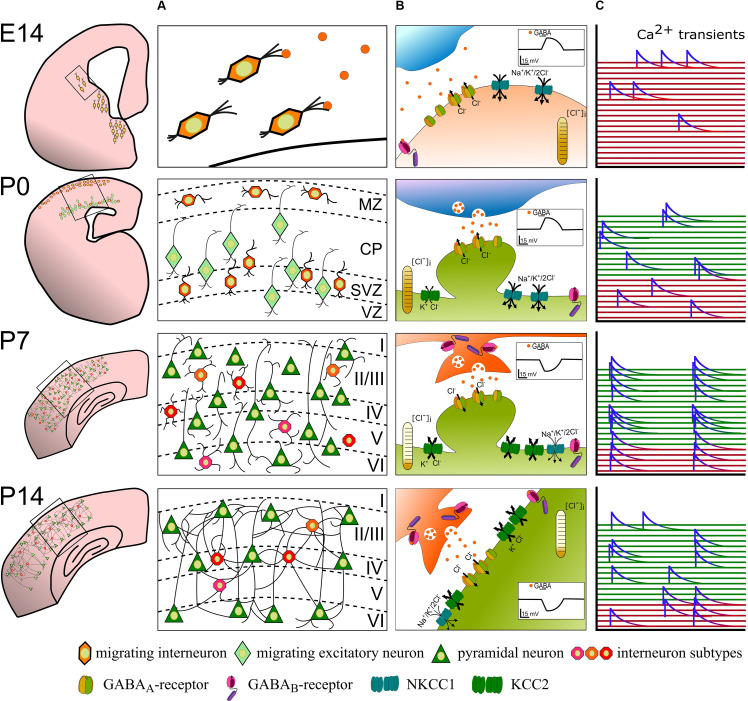
The developmental sequence of perinatal changes in **(A)** positioning and integration of nascent interneurons, **(B)** synaptic GABA signaling, and **(C)** interneuron participation in network activity in the rodent cortex. At E14, GABAergic interneuron precursors tangentially migrate from the ganglionic eminences into the cortex and ambient GABA acts as a chemoattractant. Binding of GABA to GABA_A_-receptors at this stage depolarizes recipient interneurons and likely contributes to sporadic, uncorrelated Ca^2+^ transients (red). Around birth (P0), immature GABAergic interneurons are found in the marginal zone and migrate radially concomitant with pyramidal neurons. Here, GABA acts as a Stop and Go signal mainly through GABA_A_-receptors. At this developmental stage, GABA is still excitatory and contributes to uncorrelated Ca^2+^ transients in GABAergic interneurons (red traces) and immature pyramidal neurons (green traces). At the end of the 1st postnatal week (P7), layering of the cortex is mostly complete and maturing neurons already begin to form synapses. Vesicular released GABA hyperpolarizes the postsynaptic cell *via* postsynaptic GABA_A_- and binds to extra- and pre-synaptic GABA_B_-receptors. Highly synchronous spontaneous activity in interneurons and pyramidal neurons is a hallmark of this developmental phase. By P14, excess neurons were depleted through developmental apoptosis and stable functional, also perisomatic connections have developed. The cation-chloride transporter profile is fully matured and GABA receptor signaling fulfills its mostly inhibitory function. Spontaneous and sensory-evoked activity shifts to a more decorrelated state.

Before becoming the main inhibitory neurotransmitter in the mature brain, GABA exerts mainly excitatory function in immature neurons (Luhmann and Prince, 1991; Leinekugel et al., 1995; Rheims et al., 2008; Kirmse et al., 2015) and is suggested to regulate spontaneous activity during development (Ben-Ari, 2002; Le Magueresse and Monyer, 2013; Kirmse et al., 2015). In turn, the neuronal activity itself is a key regulator of the subcellular processes that underlie this developmental change in GABA signaling—like the expression of chloride transporters (Fiumelli et al., 2005), GABA-receptor expression, and GABAergic synaptogenesis (Ganguly et al., 2001; Wardle and Poo, 2003; see also Figure 1 for overview).

### GABA-Receptor Signaling

At the postsynaptic side, GABA exerts its function *via* ionotropic GABA_A_-receptors and metabotropic GABA_B_-receptors. GABA_A_-receptors are a heterogenous group of chloride channels with rapid kinetics. Each of them is formed by a heteromeric complex that consists of five of a possible 19 different subunits (α1–6, β1–3, γ1–3, π, θ, δ, ε, Kumada and Fukuda, 2020). Differences in localization of GABA_A_-receptors lead to two major forms of GABAergic inhibition: phasic inhibition and tonic inhibition. Where the former is mediated by synaptic, low affinity GABA_A_-receptors, the latter is facilitated by high affinity, extrasynaptic GABA_A_-receptors (Kumada and Fukuda, 2020). The expression of GABA_A_-receptor subunits changes with development: some subunits display a consistent increase in expression levels with age (e.g., α1, β1, β2, δ) whereas others instead show a peak, followed by a decline (e.g., α2, α3, α5, β3, γ3, Laurie et al., 1992). Although a large portion of GABA_A_ receptor subunits is only found in postmigratory neurons, others (α2, α3, β1, β3, γ1, γ2) are already detectable in the germinal zone and in migrating neurons in the marginal zone and the cortical plate (Araki et al., 1992; Laurie et al., 1992; Poulter et al., 1992; Van Eden et al., 1995). The early timing of the expression profile supports an effective role of GABAergic signaling before synaptogenesis, i.e., during interneuron migration and maturation of GABAergic synapses.

In contrast to the ionotropic GABA_A_-receptors, the metabotropic GABA_B_-receptors consist of two distinct subunits (B1 and B2). Subunit B1 is expressed in two isoforms: namely, B1a and B1b, which require the dimerization with a B2 subunit to form functional heteromeric GABA_B_-receptors (Terunuma, 2018). Once the receptor is activated by ligand binding on the extracellular domain of the B1 subunit, a G-protein mediated signaling cascade is started which opens K^+^ channels at the post- and Ca^2+^ channels at the presynaptic site. In this way, GABA_B_ receptor mediated inhibition leads to hyperpolarization of the postsynaptic neuron and/or to reduced release probability of neurotransmitters in the synaptic cleft. In rodents, GABA_B_ receptors are expressed as early as embryonic day 14 (López-Bendito et al., 2002) and reach their expression level peak in the first postnatal week (Turgeon and Albin, 1994; Behuet et al., 2019). Furthermore, it was shown that the different GABA_B_ subunits have distinct expression levels with GABA_B1_ playing a more important role during prenatal development of the rat (Li et al., 2004). In addition, GABA_B1_-receptors are expressed in migrating neurons in the lower intermediate zone, where GABA not only enhances GABA_B_-receptor expression but also works as a chemo-attractant that promotes motility of migrating neurons (Behar et al., 2001). GABA_B_ receptors are found in dendritic spines and dendritic shafts at extrasynaptic and perisynaptic sites during postnatal development (López-Bendito et al., 2002). Moreover, in the postnatal stage, activity-dependent secretion of brain-derived neurotrophic factor (BDNF) is mainly mediated by activation of GABA_B_-receptors, which then promote the development of perisomatic GABAergic synapses (Fiorentino et al., 2009).

Taken together, the results on GABA receptor signaling during brain development illustrate the importance of GABA_A_-receptor activity for corticogenesis, interneuron migration, and for modulation of synaptic transmission (Cancedda et al., 2007; Patrizi et al., 2008; Fuchs et al., 2013) and indicate a potentially important but largely unresolved role for GABA_B_-receptors.

### Facilitating the Chloride Gradient: The Cotransporters NKKC1 and KCC2

Activation of mature postsynaptic GABA_A_ receptors typically leads to a fast hyperpolarization through anion influx, predominantly by Cl^−^ (Kaila, 1994; Olsen and Sieghart, 2009). However, during brain development, GABA plays a critical role as an excitatory drive relevant for the proper development and establishment of neuronal circuits (Ben-Ari, 2002; Rheims et al., 2008). In fact, in the immature brain GABA_A_-receptor activation leads to depolarization of neurons due to the high intracellular Cl^−^ concentration (Rivera et al., 1999; Yamada et al., 2004; Rheims et al., 2008; Kirmse et al., 2015). The intracellular concentration is mostly set by two main cation-chloride cotransporters Na^+^-K^+^-2Cl^−^-Cotransporter 1 (NKCC1)—a chloride-importer—and K^+^-Cl^−^-cotransporter 2 (KCC2)—a chloride extruder, which play a pivotal role in the polarity of GABAergic action (Rivera et al., 1999; Yamada et al., 2004; Achilles et al., 2007; Rheims et al., 2008; Kirmse et al., 2015). In immature cortical neurons, intracellular chloride is significantly higher than in mature neurons due to the predominant expression of NKCC1 over KCC2. The developmental change in chloride-cotransporter expression, which occurs within the first postnatal week in the rodent cortex (Shimizu-Okabe et al., 2002), is hence effectively reversing GABA action from depolarizing to hyperpolarizing (Rivera et al., 1999; Shimizu-Okabe et al., 2002; Yamada et al., 2004; Rheims et al., 2008). Studies in various animal models have shown that this switch occurs at different time points within different species and have brain region-specific effects (Leinekugel et al., 1995; Reith and Sillar, 1999; Saint-Amant and Drapeau, 2000; Eilers et al., 2001; Gao and Van Den Pol, 2001; Murata and Colonnese, 2020). It also could be demonstrated that the precise time point of the switch is not strictly determined by the genetic program, but might be influenced by neurotrophic factors and neuronal activity (Ganguly et al., 2001; Wardle and Poo, 2003). For example, repetitive fast postsynaptic excitation influences KCC2 expression and therefore affects the chloride reversal potential (Fiumelli et al., 2005). Also, GABA itself can be crucial for the determination of the time point of shift. In the turtle retina, under blockade of GABA_A_ receptors at the developmental time point of the shift, GABA action remains excitatory, through inhibition of KCC2 upregulation (Leitch et al., 2005). On the other hand, experiments in hippocampal slice and dissociated hippocampal cultures do not support a GABA and/or activity dependency of the switch from de- to hyperpolarizing (Ludwig et al., 2003; Titz et al., 2003). Unfortunately, studies aiming to assess the role of activity for the expression of NKCC1 are still difficult to interpret, likely because of broad technical difficulties (Virtanen et al., 2020). A recent study, in which NKCC1 is selectively knocked-out in telencephalic glutamatergic neurons, showed that in the visual cortex NKCC1 is not necessary for the establishment of fully functional networks in adult mice (Graf et al., 2021). Conceptually this is in line with another recent study, in which GABAergic activation did not produce excitation in postsynaptic neurons in the visual cortex of 3 days old mice (Murata and Colonnese, 2020). Supporting brain region specific differences in GABAergic synaptic transmission, glutamatergic hippocampal neurons lacking NKCC1 display significantly lower intracellular chloride concentrations. Despite the alterations in correlated spontaneous activity during development and slightly altered network dynamics in the hippocampus of adult mice, these knock-out mice are perfectly capable of performing hippocampus-dependent behavioral tasks (Graf et al., 2021). However, it remains unclear whether changes in network dynamics are an acute effect of NKCC1 loss, or rather an adaptation to ensure proper functionality in NKCC1 knock-out mouse lines. In support of the latter hypothesis, an earlier study showed that the complete loss of NKCC1 prevents excitation *via* GABA in hippocampal CA3 neurons, nevertheless, these mice still display typical network activity patterns as seen under physiological conditions (Sipilä et al., 2009). In contrast, another constitutive NKCC1 knock-out mouse line shows impairments in early hippocampal activity patterns and delayed maturation of the network (Pfeffer et al., 2009).

### GABAergic Signaling Before and During Synaptogenesis

On a structural level, GABAergic synapses are among the first synapses that are formed in the developing brain (Tyzio et al., 1999; Khazipov et al., 2001; Rymar and Sadikot, 2007). Immature neurons in the hippocampus as well as in the neocortex first receive GABAergic before glutamatergic input (Ben-Ari, 2006; Wang and Kriegstein, 2008). In the neocortex of newborn mice GABAergic vesicle abundance is relatively low and only during the following days the expression of GABAergic synaptic markers increases gradually until it reaches a plateau at the end of the 2nd postnatal week (Minelli et al., 2003). However, not only does the number of GABAergic vesicles increase, but also their overall distribution changes within the developing cortex. While GABAergic vesicles can only be detected in the marginal zone in newborn mice, their distribution gradually extends deeper into the neocortex until finally covering all cortical layers at the end of the second postnatal week (Minelli et al., 2003). Despite the maturation of GABAergic vesicles late in the first postnatal week (Minelli et al., 2003), GABA positive cells can already be found even in the deeper layer of the neocortex at birth (Takayama and Inoue, 2010). These findings support a role of GABAergic signaling before the onset of synaptogenesis, i.e., extrasynaptic transmission. In line with this, GAD67 (the main GABA-producing enzyme isoform) and GABA_A_ receptors can already be detected as early as E17 in the ventricular zone (Ma and Barker, 1995) and throughout the cortical plate (van den Berghe et al., 2013). Paracrine release of GABA was demonstrated to occur in different cell types during development, e.g., in immature neurons, but also in endothelial cells (Taylor and Gordon-Weeks, 1991; Gao and Van Den Pol, 2000; Li et al., 2018). In the latter, partial or complete loss of GABA release during embryogenesis leads to impairment of long-distance migration and positioning of cortical interneurons (Li et al., 2018). In the adult cortex, astrocytes express the GABA transporter GAT1 and thus influence the excitatory and inhibitory transmission through the paracrine spread of GABA (Minelli et al., 1995; Barakat and Bordey, 2002). However, whether or not astrocytes are also a source of GABA during development is yet not clear.

### Maturation of GABAergic Synapses

Neuronal activity e.g., *via* the depolarization of immature neurons, is a key regulator in synaptogenesis. *in vitro* and *in vivo* studies show that the excitatory effect of GABA during early development is essential for the normal maturation of dendritic spines (Hensch et al., 1998; Cancedda et al., 2007; Chattopadhyaya et al., 2007; Wang and Kriegstein, 2008; Pfeffer et al., 2009; Oh et al., 2016; Flossmann et al., 2019). In line with an important role for GABA_A_-receptor-mediated activity during the establishment of neural circuits, the development of synapses between somatostatin-positive (SST) interneurons and pyramidal cells in the hippocampus is NKCC1-dependent (Pfeffer et al., 2009; Flossmann et al., 2019). However, not only GABA-induced activity is required for the proper maturation of GABAergic interneurons, but also, NMDA receptor activity affects the regulation of GABAergic synaptogenesis (Cserép et al., 2012; Gu et al., 2016; Hanson et al., 2019). Tonic NMDA-mediated neuronal activity is important for the maturation and correct integration of parvalbumin-positive (PV) interneurons into the developing cortical network (Hanson et al., 2019). In early development, NMDA-receptors are co-localized with GABA_A_-receptors at the postsynaptic site (Cserép et al., 2012), where NMDA-receptors act as upstream signaling molecules essential for GABAergic synaptogenesis *via* Ca^2+^ transient and calmodulin signaling (Gu et al., 2016). Conversely, GABA_A_-receptor activation is sufficient to remove the voltage-dependent Mg^2+^ blockade and thus activate NMDA-receptors (Wang and Kriegstein, 2008). The mutual interplay between GABA_A_- and NMDA-receptors is thus shown to play an important role in the emergence of spontaneous synchronous activity and the correct balance between excitation and inhibition (E/I) in the neocortex (Wang and Kriegstein, 2008). Of note, also AMPA-receptor expression at the postsynaptic site can be affected by GABAergic action, such that AMPA-receptor levels are downregulated in glutamatergic/GABAergic-mixed synapses (Fattorini et al., 2019). Thereby, a proper E/I balance is ensured and a potential neuroprotective effect is exerted in the developing brain (Fattorini et al., 2019). GABA_A_-receptors fulfill important functions for GABAergic synapse development not only on the functional but also on the structural level (Chattopadhyaya et al., 2007; Deng et al., 2007; Fuchs et al., 2013; Oh et al., 2016). Along this line, GABA release from SST interneurons leads to the expression of the scaffolding protein gephyrin and dendritic spine formation by recruitment and activation of GABA_A_ receptors in layer 2/3 cortical pyramidal neurons in neonatal mice (Oh et al., 2016). Accordingly, conditionally knocking-out GAD67 in PV basket cells results in less terminal branching, smaller boutons size, and hence, fewer and deficient synaptic contacts (Chattopadhyaya et al., 2007). Depletion of GAD65, the smaller isoform of the GAD protein, leads to impaired formation of cortical networks and over-responsiveness in the visual cortex (Hensch et al., 1998) while overexpression of GAD67 leads to faster perisomatic innervation (Chattopadhyaya et al., 2007). Together, these findings suggest that GABA regulates perisynaptic contact formation during the maturation of neural circuits (Chattopadhyaya et al., 2007) and imply that suppression of electrical activity leads to fewer synaptic contacts *via* reduced GABA levels (Chattopadhyaya et al., 2004). On the other hand, mice with disturbed GABA homeostasis also display less activity (Fiorentino et al., 2009). This raises the question of whether GABA action on synaptogenesis should be mostly considered as an activity-independent mechanism.

## The Interplay of Activity and Perinatal Changes of GABAergic System at The Cellular and Network Level

**Figure 2:**
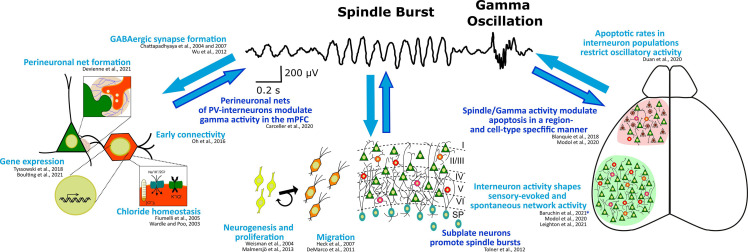
Interdependence of (oscillatory) cortical network activity and development of the GABAergic system at the subcellular, cellular, and network level. Known regulation of or by high frequency spindle burst and gamma activity are highlighted in dark blue. Exemplary references to experimental evidence supporting these processes are listed. *Note, this study suggests that somatostatin interneurons contribute to the early processing of sensory information in a way, that their activity affects spindle burst activity possibly through the plastic arrangement of thalamic innervation to the neocortex.

Not only the maturation of the GABAergic system at the subcellular level is affected by, but also the maturation of the single (inter-)neuron and network level activity shows an activity-dependence. While, on the other hand, network composition in general—and especially the activity of GABAergic subpopulations—significantly influence cortical activity during the postnatal period of rodents (Le Magueresse and Monyer, 2013; Kepecs and Fishell, 2014; Tremblay et al., 2016; for an overview see also Figure 2). Inversely, cortical activity is not only the most relevant cortical output function, but also has an important feedback role as a key regulating factor for many processes at the cellular and network level during early brain development (Luhmann et al., 2016; Okujeni and Egert, 2019). In this way (and as can be seen in Figure 2), cortical activity and especially the activity of interneurons themselves critically control several key steps in the development of GABAergic neurons on the network level, including migration, wiring, and programmed cell death.

### Neurogenesis and Proliferation of Interneuron Precursors

In chronological order, the first step to consider is the embryonic generation of GABAergic interneurons, i.e., the neurogenesis and proliferation of interneuron precursor cells in the ventral telencephalon—in particular the medial and caudal ganglionic eminences (MGE and CGE, respectively), with a minor contribution of the preoptic area and the lateral ganglionic eminence (POA and LGE; Gelman and Marín, 2010; Sultan et al., 2013). Expression of homeobox transcription factors of the Dlx family is of essential importance for GABAergic precursors proliferation, as well as for the differentiation of interneurons (Petryniak et al., 2007). Additionally, both processes are activity-dependent. The proliferation of neuronal progenitors, in general, has been shown to be influenced by spontaneous calcium activity (Weissman et al., 2004; Malmersjö et al., 2013). Meanwhile, spontaneous calcium activity in parallel also critically impacts the further specification of neuronal phenotypes (Ciccolini et al., 2003; Borodinsky et al., 2004), which is no surprise considering the tight link between neuronal gene expression and neuronal activity (Flavell and Greenberg, 2008). In this regard, it should be highlighted that the increasing complexity of activity patterns in developing neurons that follows the occurrence of simple calcium transients in progenitor cells also offers a higher order complexity on the level of gene regulation (Tyssowski et al., 2018). Activity-dependent regulation of the proliferation and differentiation of neural stem cells and oligodendrocyte precursors has also been shown in the postnatal brain (Káradóttir and Kuo, 2018). Thus, neuronal activity is not only an import modulator determining the extent and type of interneurons during development but also remains important in adult neuro- and gliogenesis. Moreover, *via* the direct action of synaptically released GABA (Andäng et al., 2008) as well as through cortical activity that is in turn significantly influenced by GABAergic neuronal population sizes (Modol et al., 2020), cross-talk of interneuron proliferation and cortical activity should be carefully considered as a regulatory mechanism that shapes neuronal circuitry.

### Migration of Interneurons

Upon their generation, interneurons need to migrate from their places of origin in the subpallium along the subventricular and marginal zone to reach their final place of destination in the postnatal cortex. This location is spatially characterized by a distinct radial position within a certain cortical region and also a distinct laminar location within a certain cortical layer (Faux et al., 2012). Early experiments *in vitro* have already shown that migration of immature neurons is generally dependent on spontaneous calcium activity (Komuro and Rakic, 1996; Komuro and Kumada, 2005). These experiments were later confirmed *in vivo*, where the pharmacological or genetic reduction of activity also altered the migration of excitatory and inhibitory neurons (Heck et al., 2007; De Marco García et al., 2011). In addition to this effect of neuronal activity on migration, one has to note that various neurotransmitters—including GABA—act as chemoattractants for targeting migratory streams and thus, can directly modulate neuronal migration (Behar et al., 2000; Inada et al., 2011). Since GABA release itself is regulated in an activity-dependent manner, this implies a further level of regulation on account of this (Luhmann et al., 2015). Further, more recent studies show that silencing neuronal activity e.g., by the overexpression of the Kir2.1 channel, results in mispositioning of specific interneuron subpopulations by affecting the expression of Dlx genes (De Marco García et al., 2011). This suggests that genetic programs initiated at the progenitor stage are modulated during development by activity (Bando et al., 2016; Hurni et al., 2017). Nevertheless, the experimental disentangling of direct causal links between activity and differentiation, migration, and/or integration of interneurons remains challenging and requires the careful analysis of subtype-specific differences in these relationships (Bugeon et al., 2021). Moreover, the simultaneous maturation of the inhibitory GABAergic response from its immature excitatory function (Ben-Ari, 2002) with the migration of interneurons adds another layer to the complex and multidimensional regulation needed for the correct laminar positioning of interneurons. The importance of which is also experimentally supported by the halt of neuronal motility induced by upregulation of KCC2 or pharmacological interference with GABA_A_ receptor function (Heck et al., 2007; Bortone and Polleux, 2009).

Thus, activity on the single interneuron level but also on the network level critically regulates the migration of immature neurons. However, the extent to which spatial and temporal changes in the migration of interneurons impacts cortical activity and function needs further investigations, especially since interneuron migration is tightly linked to the specification of interneurons (Lim et al., 2018b).

### Specification of Interneuron Subtypes

In the neocortex, the vast majority of GABAergic cells are represented by local circuit interneurons, which are traditionally classified as aspiny neurons (Lodato and Arlotta, 2015). All GABAergic interneurons produce GABA, the hyperpolarizing action of which in the mature brain accounts for their definition as inhibitory neurons. GABAergic neurons form a heterogeneous population, of which classification is an ongoing effort that encompasses several morphological, electrophysiological, molecular, connectivity, and transcriptomic properties (Kepecs and Fishell, 2014). The broadest and most widely adopted classification relies on molecular markers, two of which (namely: parvalbumin and somatostatin) label around 70% of cortical interneurons. The remaining 30% are instead identified by a handful of markers, among which the most prevalent one is the serotonin receptor 5hT3aR. Other markers—such as the vasointestinal peptide (VIP), reelin (RELN), cholecystokinin (CCK), and calretinin (CR)—label smaller subclasses.

The maturation of subtype-specific properties of inhibitory interneurons mainly occurs during the first weeks of postnatal development in rodents and the different types of interneurons become only observable after the migration is complete at the end of the 1st postnatal week (Lim et al., 2018b). Whether the lineage specification is already predetermined during the embryonic stage or is (partially) acquired during the postnatal period through a microenvironment-mediated influence is still a matter of debate (Wamsley and Fishell, 2017; Lim et al., 2018a). However, it is becoming evident that activity-dependent mechanisms impinge on cellular properties of interneurons, such as morphology, synapse specificity, and connectivity (De Marco García et al., 2011; Dehorter et al., 2015). Indeed, many supporting findings are coming out from studies that manipulate or abolish the activity of certain cell-type precursors (MGE or CGE derived) (Chattopadhyaya et al., 2004, 2007; De Marco García et al., 2011). Some of these findings suggest an activity-dependent regulation of molecular and electrophysiological properties of different interneuron subtypes (Miller et al., 2011; Dehorter et al., 2015) and thereby contribute to subtype-specific differences in gene expression (Batista-Brito et al., 2008; Paul et al., 2017), which have now been resolved with increasing depth (Joglekar et al., 2021; Scala et al., 2021). Furthermore, the onset and duration of these activity modulations have differential effects on the different interneurons subtypes, reflecting the timeline with which they differentiate from the respective ganglionic eminences (Wamsley and Fishell, 2017). Mostly early maturational aspects of MGE- and CGE-derived interneuron specification are hereby discussed, since general morphological and electrophysiological characteristics that distinguish the different interneuron subpopulations are extensively described elsewhere (Gelman and Marín, 2010; Rudy et al., 2011; Pfeffer et al., 2013; Lim et al., 2018a; Fishell and Kepecs, 2020).

#### Early Maturation of MGE-Derived Interneurons

In the rodent cortex, the earliest developing interneurons are SST and PV interneurons originating from the MGE. As mentioned before, the first GABAergic neurons start to populate the cortical plate very early in development, already between E9.5 and E12.5 in deep cortical laminas (Miyoshi et al., 2007). At first, SST interneurons are generated from the dorsal division of MGE. Around E17.5, they are found in the deep layer of the cortical plate (Miyoshi and Fishell, 2011), whereas at the end of the 1st postnatal week, they are visible across all cortical layers (Liguz-Lecznar et al., 2016). During early postnatal days, SST interneurons play transient and instrumental functions in shaping neural circuits in the cortex. At P4–6 in the mouse somatosensory cortex, SST interneurons receive dense innervation from the thalamus, and in turn, give inputs to pyramidal cells, spiny stellate cells, and recently migrated prospective PV neurons (Marques-Smith et al., 2016; Tuncdemir et al., 2016). Remarkably, these connections are fundamental for the proper formation of thalamo-cortical feedforward circuits (Tuncdemir et al., 2016), the coordinated activation of PV cells (Modol et al., 2020), and functional topography (Duan et al., 2020), since conditional ablation or silencing of SST neurons drastically impairs these processes. Conversely, it has been shown that the functional maturation of SST interneurons is delayed if afferent excitatory inputs from pyramidal neurons are decreased at early (P1) rather than later (P8) postnatal stages (Pan et al., 2019). Furthermore, besides direct synaptic inputs, it was recently shown that SST interneurons also exert a paracrine role through the release of synaptogenic extracellular matrix proteins such as collagen XIX (Su et al., 2020). During the 2nd postnatal week, SST interneurons are involved in the control of sensory-evoked activity, such as spontaneous retinally-driven activity in the visual cortex (Leighton et al., 2021), or multi-whisking activity in the barrel cortex (Kastli et al., 2020). In line with these findings, conditional silencing of SST interneurons leads to a decrease in spontaneous spindle-burst activity and abolished facilitation in sensory adaptation (Baruchin et al., 2021).

Later on, PV neurons originate from the ventral division of MGE (Bandler et al., 2017) and start to radially migrate between E18 and P2, not reaching their final position until P6 (Bartholome et al., 2020). Between the 2nd and the 4th week, cortical PV neurons start expressing PV and defining ion channel composition that characterizes their peculiar electrophysiological properties (Bartholome et al., 2020). Upon arrival into the cortical layer, a particular type of extracellular matrix dubbed the perineuronal net (PNN), plays a critical role in the correct settling of PV interneurons by influencing their connectivity. The PNN can control synaptic plasticity by preventing spine formation (Vo et al., 2013). In support of this, degradation of PNNs leads to reduced gamma activity in juvenile mice (Carceller et al., 2020), which is in line with recent discovery linking PV to gamma activity (Bitzenhofer et al., 2020) and the finding that altered PNNs lead to abnormal activity (Wingert and Sorg, 2021). In addition to the role of PNNs, other molecular mechanisms can influence PV development, such as tonic activation of NMDA receptor (Hanson et al., 2019), BDNF (Lau et al., 2021), or retinoic acid (Larsen et al., 2019), whose receptor expression is also dependent on activity.

#### Early Maturation of CGE-Derived Interneuron

In the rodent brain, CGE-derived interneurons are produced at first at E12.5, reaching a peak around E16.5 (Miyoshi et al., 2010). Unlike MGE-derived cortical interneurons, they do not populate the cortex in an inside-out manner, but the vast majority are located in superficial cortical layers, and only acquire their final position around P4 (Miyoshi et al., 2010). Remarkably, the integration into the neocortex of CGE-derived interneurons depends on serotonin signaling (Murthy et al., 2014): impairment of which leads to their mispositioning (Frazer et al., 2015). Although it has been shown that a common feature of most, if not all, CGE-derived interneurons is the expression of the serotonin ionotropic receptor 5HT3aR (Lee et al., 2010) most of our understanding nonetheless remains built upon traditional molecular markers that identify specific subclasses (Tremblay et al., 2016). Of these, the best characterized is probably the VIP interneuron subclass, which accounts for around 40% of all CGE-derived interneurons, and the reelin subclass which labels around 60% of them (Wamsley and Fishell, 2017). However, our knowledge on the early developmental phases of CGE-derived interneurons is still limited and, only recently, CGE-specific transcriptional factors and activity-dependent mechanisms began to be explored (De Marco García et al., 2011; Miyoshi et al., 2015; Wei et al., 2019). Of note, it was shown that Prox1 is fundamental for the acquisition of CGE-derived interneuron properties both in the embryonic and postnatal stage (Miyoshi et al., 2015), with its conditional knock-out during early postnatal days leading to impairment of excitatory inputs onto the VIP multipolar subtype (Stachniak et al., 2021). Remarkably, it has been shown that network activity critically affects the proper morphological development of CR-positive VIP bipolar cells and RELN interneurons, but not that of CCK-positive VIP multipolar interneurons (De Marco García et al., 2011, 2015). Thus, activity and genetic program might act in a subtype-specific manner onto CGE-derived interneuron developmental steps. Finally, with the introduction of subtype-specific driver Cre-lines early functions and regulatory mechanisms have also begun to be studied in more depth (Taniguchi et al., 2011). In the barrel cortex, for example, VIP interneurons show a transient preferential response to multi-whisking that is lost during the 3rd postnatal week (Kastli et al., 2020), and their conditional silencing influence the onset of active whisking (Baruchin et al., 2021).

### Connectivity Within GABAergic Populations and Across Transient Neuronal Populations

Interneurons are not only integrated into nascent and mature cortical networks *via* chemical synapses—of which many previously discussed pre- and postsynaptic GABAergic elements critically impact the emergence of cortical activity but also *via* gap junctions which are ubiquitous in the cortex. Gap junctions form connections mainly amongst GABAergic interneurons of the same functional class, but also across functionally distinct classes in the mature and immature cortex (Peinado et al., 1993; Hatch et al., 2017). Interestingly, gap junctions are generally described to be essential for oscillatory activity (Tchumatchenko and Clopath, 2014; Pernelle et al., 2018) and bidirectional activity-dependent plasticity is shown (Haas et al., 2016). Yet, the concise contribution of electrical coupling to distinct activity patterns during peri- and postnatal development remains unknown. Integration of GABAergic interneurons into developing cortical circuits *via* chemical synapses can be measured as spontaneous and evoked GABAergic inputs onto cortical plate neurons in the rodent cortex as early as E19 and P3, respectively (Owens et al., 1999; Daw et al., 2007). Instead, functional synaptic connections between GABAergic interneurons have only been shown after P4 in the visual cortex (Pangratz-Fuehrer and Hestrin, 2011). Prior to this, transient cortical populations already show GABAergic inputs (Kilb and Luhmann, 2001; Soda et al., 2003). However, the contribution of interneurons towards GABAergic signaling to transient cell populations like Cajal Retzius neurons, or subplate neurons that precede the integration of GABAergic cells into immature but persistent cortical circuits—is the subject of ongoing research (Molnár et al., 2020). The prerequisite for the functional integration of GABAergic interneurons is the maturation of their electrophysiological as well as their morphological features at the presynapse, but also the maturation of GABAergic synapses on the postsynaptic side of the recipient cells. This includes the aforementioned expression of GABAergic receptors and the setting of chloride and bicarbonate gradients. As discussed above, this structural and functional maturation of the GABAergic synapse occurs in an largely activity-independent manner (le Magueresse et al., 2011). Not only does neuronal activity influence the initial formation of perisomatic synapses by interneurons (Chattopadhyaya et al., 2004), but it also remains a key influencer of plastic changes on the structure and function of GABAergic synapses in the adult brain (Flores and Méndez, 2014). On the other side of the coin, many important key cortical functions depend on the proper integration of GABAergic interneurons into the cortical network, like selectivity of sensory modalities, gain control, range modulation and plasticity of cortical circuits, regulation of firing rates and bursting activity with high temporal precision, generation and synchronization of cortical rhythms, as well as the maintenance of the excitatory and inhibitory balance (Tremblay et al., 2016; Fishell and Kepecs, 2020).

### Developmental Apoptosis

Besides genetic programs, trophic support, and pro- and anti-apoptotic factors, neuronal activity also has a major impact on cell death and survival rates in the developing cortical network (Blanquie et al., 2017a; Wong and Marín, 2019). Here, increases in neuronal activity are associated with elevated survival rates in principal neurons and interneurons, whereas blockade or attenuation of activity is generally associated with higher apoptotic rates (Ruijter et al., 1991; Ikonomidou et al., 1999; Heck et al., 2008; Southwell et al., 2012). However, cell-type-specific peculiarities exist, for example in the transient cell population of Cajal Retzius neurons, where activity even fulfills an antithetic pro-apoptotic function (Del Río et al., 1995; Blanquie et al., 2017b). Whether this effect of activity for the survival of developing neurons is controlled by a cell-autonomous process or by network-dependent mechanisms is the subject of current investigations (Southwell et al., 2012; Blanquie et al., 2017c; Wong et al., 2018). Most recent evidence suggests that not only the level of neuronal activity but also the temporal pattern of activity affects neuronal survival rates *in vivo* (Blanquie et al., 2017c) and *in vitro* (Wong Fong Sang et al., 2021). This also applies to interneurons, as different evidence supports that positive or negative alterations in network activity result in a respective change of survival rates in GABAergic interneurons (Wong et al., 2018; Duan et al., 2020; Bitzenhofer et al., 2021). Notably, the most potent neuroprotective patterns highlighted within these studies are of a high-frequency oscillatory nature and resemble activity which typically occurs at the end of the 1st postnatal week *in vivo* (Yang et al., 2009; Luhmann and Khazipov, 2018) or is reflected *in vitro* by reminiscent patterns such as recurrent bursts (Wagenaar et al., 2006; Sun et al., 2010). Interestingly, GABAergic neurons themselves are essential for the modulation of these cortical activity patterns (Bonifazi et al., 2009; Isaacson and Scanziani, 2011; Modol et al., 2020). Thus, as far as the understanding of the mutual dependency of activity and apoptosis in interneurons goes until now, cortical activity acts as a master regulator of apoptotic rates in both interneurons and pyramidal neurons (Wong et al., 2018), even in a region-specific manner (Blanquie et al., 2017c). Herewith, activity-dependent regulation of developmental cell death can be seen as a* bona fide* homeostatic system (Blanquie et al., 2017a; Causeret et al., 2018) with the GABAergic interneurons in the perfect position to orchestrate this cortical activity set point (Duan et al., 2020).

### How Do Dynamic Changes in the GABAergic Neuron Fraction During Perinatal Development Affect Network Activity in the Developing Cortex?

The sequential generation, migration, and apoptotic removal of interneurons during early brain development eventually influence GABAergic population sizes in the mature cortex, but also cause a dynamic variation in the absolute GABAergic neuron population size in the cortex during the developmental phase. Yet, the relative GABAergic neuron fraction is maintained throughout the embryonic and postnatal development and into adulthood (Sahara et al., 2012). Experimental manipulations of excitation/inhibition ratio are effectively compensated for, either through adjustments in the number of connections (Sukenik et al., 2021) or changes in synaptic strength (Southwell et al., 2012). Similar adaptive mechanisms also stabilize cortical inhibition on the network level under physiological (Southwell et al., 2012; Field et al., 2020; Romagnoni et al., 2020) and pathophysiological conditions (Hunt et al., 2013). Thus, in line with the dispensability of NKCC1-mediated depolarizing GABA responses for the establishment of cortical activity patterns (Graf et al., 2021), cortical networks adapt surprisingly well to alterations in the relative GABAergic fraction (Liu, 2004; Sukenik et al., 2021) and thereby keep the network activity level and patterning mostly stable. Both phenomena—specifically the stable expression of network activity despite the physiological changes in absolute GABAergic population during development, but also the tight homeostatic regulation of activity upon pathological or experimental perturbations of the GABAergic system—emphasize the importance of network activity as the most relevant output function. At the same time, these findings do not exclude that the developmental changes in interneuron function and network composition cause *per se* physiologically relevant difference in this output, i.e., merging network activity patterns throughout development and differences in activity patterns across models (Luhmann et al., 2016). Deciphering the multi-layered developmental processes in GABA signaling discussed above is necessary for the future assessment of the exact contribution of these processes to cortical activity patterns seen during development and in adult cortical networks. Certain partly-transient network structures, such as clustered GABAergic assemblies (Tuncdemir et al., 2016; Modol et al., 2020), subplate neurons (Kanold and Shatz, 2006; Molnár et al., 2020), and subcortical thalamic regions (Minlebaev et al., 2011; Yang et al., 2013; Murata and Colonnese, 2016), are surely essential and thus not dispensable for the establishment of cortical network activity and function during early brain development (Tolner et al., 2012).

## Conclusion and Outlook

Immature cortical networks have a unique capacity to stabilize their network activity, even if strong changes in GABA signaling are introduced e.g., by alterations in the absolute number of GABAergic interneurons in neocortical cultures (Sukenik et al., 2021; Xing et al., 2021), genetic changes of total GABA content in the brain (Tamamaki et al., 2003), or modulations of chloride homeostasis (Pfeffer et al., 2009; Graf et al., 2021). This stability underlines the great source of plasticity of the neuronal system in general, but is especially remarkable given the suggested key function of GABAergic interneurons for the balancing of excitation and inhibition, and thus coordinating network activity during development (Bonifazi et al., 2009; Le Magueresse and Monyer, 2013; Modol et al., 2020; Baruchin et al., 2021). By and large, GABAergic interneurons keep this crucial role in mature networks with some critical modifications (Markram et al., 2004; Bartos et al., 2007; Tremblay et al., 2016). While it is well accepted that GABAergic neuron-mediated inhibition is essential for the regulation of synchronized oscillations in adult cortical networks (Klausberger and Somogyi, 2008; Gonzalez-Burgos et al., 2010), the functional role of interneurons during development is still less clear. It remains to be seen, if the activity of distinct interneuron subclasses during development is crucial *per se* for brain development, as suggested by recent studies (Modol et al., 2020; Baruchin et al., 2021; Leighton et al., 2021), or if only certain network activity patterns must be played in distinct cortical compartments or temporal windows for proper brain development-regardless of the GABAergic contribution. Interestingly, a prolonged developmental timeline for GABAergic interneurons is an amplified trait in higher order gyrencephalic mammals, which suggests that a protracted development of interneurons through neurogenesis, neuronal migration, and network integration is a mechanism for increased complexity and cognitive flexibility in cortex function (Kim and Paredes, 2021).

In view of the above, the association of pathophysiological changes in interneuron function or excitation/inhibition balance with neurological and psychological conditions in humans are to be expected and have been well described (Marín, 2012; Nelson and Valakh, 2015). With pharmacological GABAergic modulators such as benzodiazepines as first-line treatment options in acute epileptic emergencies in children and adults (Glauser et al., 2016), the direct intervention with GABA_A_ receptor signaling is already common practice in the clinic and will likely profit from future advances in this field of research. Additionally, the absence of certain activity patterns during critical developmental periods, to which GABAergic interneurons significantly contribute, is associated with unfavorable outcomes in humans and animal models (Ranasinghe et al., 2015; Whitehead et al., 2016). Thus, scientific progress will likely also provide important insights to the clinically relevant questions: (I) how pre- and early postnatal pathophysiological insults (e.g., *in utero* inflammation/infection, perinatal hypoxia-ischemia); or (II) certain drugs that impact GABAergic signaling (e.g., medications or drug abuse during pregnancy) change spontaneous activity; (III) how these activity changes ultimately affect clinical outcomes; and (IV) which clinical interventions could be advisable (ter Horst et al., 2004; Iyer et al., 2014).

Besides the manifold developmental changes in both interneuron function and cortical activity which are described in this review, in addition to the pathophysiological changes in this mutual interaction (described in more detail elsewhere; Marín, 2012), makes it more and more evident that physiological conditions—as well as anatomical and even subcellular compartment location—critically impact the contribution of GABA signaling to neuronal activity, and* vice versa* (Raimondo et al., 2017; Düsterwald et al., 2018). While current research in this field has already begun to understand these subcellular effects of ionic plasticity (Blaesse et al., 2009) and coincidence membrane depolarization (Doyon et al., 2011; Raimondo et al., 2012) on network activity in the adult brain (Jedlička and Backus, 2006; Raimondo et al., 2017), the relevance of subcellular as well as regional or state-dependent differences in GABA signaling and their impact on cortical network activity during development, remains largely unexploited. Hence, the final portrait of interneurons as replaceable or unique orchestra members and/or designated conductors of cortical activity within the orchestra line-up of the immature cortex remains a vibrant field of research with many open questions. We are only beginning to understand: (I) how interneuron subpopulations and subcellular processes contribute to spontaneous and evoked activity patterns on the network level; (II) how the GABAergic contribution differs across functionally distinct cortical regions and converging periods of development; and (III) how cortical network activity eventually feeds back on nascent interneuron function. However, it is becoming more evident that cortical network activity should be considered as the most significant output in development or, in the figurative sense, as the most sonorous symphony that the heterogenous orchestra of the developing neocortex has to play.

## Author Contributions

DW and JS contributed equally to this manuscript. All authors contributed to the article and approved the submitted version.

## Conflict of Interest

The authors declare that the research was conducted in the absence of any commercial or financial relationships that could be construed as a potential conflict of interest.

## Publisher’s Note

All claims expressed in this article are solely those of the authors and do not necessarily represent those of their affiliated organizations, or those of the publisher, the editors and the reviewers. Any product that may be evaluated in this article, or claim that may be made by its manufacturer, is not guaranteed or endorsed by the publisher.
